# M1 Macrophages Induce PD-L1 Expression in Hepatocellular Carcinoma Cells Through IL-1β Signaling

**DOI:** 10.3389/fimmu.2019.01643

**Published:** 2019-07-16

**Authors:** Zhaoyun Zong, Jiahuan Zou, Rudi Mao, Chao Ma, Na Li, Jianing Wang, Xiaoyan Wang, Huaiyu Zhou, Lining Zhang, Yongyu Shi

**Affiliations:** ^1^Key Laboratory of Infection and Immunity of Shandong Province, Department of Immunology, School of Basic Medical Science, Shandong University, Jinan, China; ^2^Department of Pathology, Qilu Hospital of Shandong University, Jinan, China; ^3^Department of Parasitology, School of Basic Medical Science, Shandong University, Jinan, China

**Keywords:** hepatocellular carcinomas, PD-L1, CD274, B7-H1, macrophages

## Abstract

Hepatocellular carcinoma (HCC) is a prototype of inflammation-related cancer, harboring M1-like and M2-like tumor-associated macrophages. M1 macrophages are thought to be tumoricidal, but some studies report its pro-tumor role. The programmed cell death-ligand (PD-L) 1 expressed in HCC cells is a critical checkpoint molecule to mediate immune escape of HCC. The PD-L1 expression in HCC cells is inducible. In the present study, we ask whether M1 macrophages induce the expression of PD-L1 in HCC cells. First, an association between M1 macrophage infiltration and PD-L1 expression in HCC tissues was determined by bioinformatics and immunohistochemistry experiments. The enrichment score of M1 macrophages was correlated to PD-L1 expression in 90 HCC samples from GEO database. Besides, infiltration of CD68+HLA-DR+ M1-like macrophages correlated with PD-L1 expression level in HCC cells. Moreover, M1-conditioned media was prepared from M1 macrophages derived from THP-1 cell, RAW264.7 cell or murine bone marrow. These supernatants induced expression of PD-L1 in HCC cells. Furthermore, inflammatory cytokine IL-1β in the supernatants was identified to account for the inducible PD-L1 expression by siRNA assay and receptor blockade assay. Additionally, transcription factor p65 and IRF1 in the HCC cells were revealed by CHIP assay to mediate the inducible PD-L1 expression. All the results demonstrate that M1 macrophages induced expression of PD-L1 in HCC cells, supporting the pro-tumor role of M1 macrophages.

## Introduction

Development of hepatocellular carcinoma (HCC) is closely associated with inflammatory microenvironment due to viral infection, obesity or damage by aflatoxin (1). Tumor-associated macrophages (TAM) are the major constituent of the inflammatory microenvironment of HCC. In response to environmental signals, macrophages undergo activation that exists on a spectrum with two extreme states termed M1 and M2 (2). M2 macrophages are usually believed to promote tumorigenesis and tumor progression (3) while M1 macrophages are thought to be tumoricidal (4). However, accumulating evidences demonstrate that M1 macrophages also have pro-tumor functions. For instances, M1 macrophages induce epithelial-mesenchymal-transition of pancreatic ductal adenocarcinoma cells (5). Additionally, M1 macrophages enhance motility of HCC cells (6). But it is unknown whether there are other mechanisms whereby the M1 macrophages promote HCC development.

The programmed cell death-ligand (PD-L) 1/programmed cell death protein (PD)-1 pathway is a critical immune checkpoint in anti-tumor immunity. PD-L1 protein is normally expressed on immune cells and in immune privileged tissues, but its expression is upregulated in many cancers, including HCC (7, 8). PD-L1 expressed by cancer cells can engage PD-1 expressed by cancer-specific CTL, inducing apoptosis or functional exhaustion of the CTLs. The immunotherapy targeting the checkpoint achieves great success in the clinic (7, 9). The clinical therapeutic benefits make research of regulatory mechanisms of PD-L1 expression in cancer cells intriguing.

PD-L1 expression in cancer cells can be classified into inducible expression, attributed to extrinsic factors in the microenvironment, and constitutive expression, attributed to intrinsic cancer-driving gene alteration (10). PD-L1 expression in HCC cells is mainly regulated by extrinsic signals from the microenvironment since PD-L1 expression can be detected by immunohistochemistry in HCC surgical and biopsy specimens but majority of cultured HCC cell lines does not constitutively express PD-L1. Cytokine IFN-γ derived from T cells is considered as an important inducer (11). But the relationship between M1 macrophages and PD-L1 expression in HCC cells remains unknown.

In the present study, we ask whether M1 macrophages promote expression of PD-L1 in HCC cells. We found that PD-L1 expression in HCC cells was correlated to infiltration of M1-like macrophages in HCC tissues. M1 macrophages were prepared from THP-1 cells, RAW264.7 cells or murine BMDM. Supernatant from the M1 macrophage culture induced expression of PD-L1 in HCC cells. Cytokine IL-1β in the supernatant was identified to be responsible for the inducible expression of PD-L1. Moreover, transcription factor P65 and IRF1 were found to be involved in the inducible PD-L1 expression.

## Methods and Materials

### Immunohistochemistry (IHC)

Fifty-eight cases of HCC paraffin-embedded specimens were from Qilu hospital, Shandong University. All the patients gave their written informed consent. The study was proved by Medical Ethical Committee of Shandong University. Immunohistochemical double staining assay was performed with a double-staining kit (ZSGB-BIO, Beijing, China) as described in our previous study (6) to measure the expression of CD68 and HLA-DRα. The CD68 molecule is a pan-marker of macrophages and CD68^+^HLA-DR^+^ are makers of M1-like macrophages. The CD68+ TAM and CD68+HLA-DR+ M1-like TAM were counted manually at high-power fields (magnification of x 400) by microscopy. Immunohistochemistry single staining assay was performed on the section serial to the section for measurement of CD68 and HLA-DRα expression to evaluate PD-L1 expression in HCC cells. The primary antibody was a rabbit anti-human PD-L1 antibody (13684s, CST, USA). The secondary staining was performed with Elivision™super HRP IHC Kit (Maixin Co., China). A placental chorionic tissue was used as the positive control. The negative control was performed by omitting the primary antibodies.

### Bioinformatics Analysis

Ninety cases of HCC samples were selected from the data series GSE41804 (20 cases) and GSE121248 (70 cases) in the GEO database (https://www.ncbi.nlm.nih.gov/geo/). The raw data was download and analyzed in the software R studio to acquire a gene-expression matrix file for the 90 HCC samples. Then, the gene-expression matrix file was uploaded to the webtool xCell (http://xcell.ucsf.edu/)(12) to acquire enrichment scores of macrophages, M1 macrophages, M2 macrophages and CD8+ T cells in HCC tissues. Lastly, Prism 6.0 was used to analyze the correlation between the scores of these immune cell infiltration and PD-L1 expression in the 90 cases of HCC samples.

The co-expression relationship between PD-L1 and IL-1β was analyzed in the muR2: Genomics Analysis and Visualization Platform (https://hgserver1.amc.nl/cgi-bin/r2/main.cgi) with 371 cases of HCC samples from the TCGA database.

### Polarization of Macrophages and Cell Cultures

Human HCC cell lines (BEL-7402, SMMC-7721 and Huh-7), human monocytic cell line THP-1, murine HCC cell line Hepa 1-6 and murine macrophage cell line RAW264.7 were obtained from the Cell Bank of Type Culture Collection of Chinese Academy of Science, Shanghai, China. All the cells were maintained in DMEM supplemented with 10% heat-inactivated fetal bovine serum at 37°C with 5% CO2.

Polarization of macrophages derived from THP-1 cells, RAW264.7 cells or bone marrow derived macrophages (BMDMs) was diagramed in Supplementary Figure 1. The mature phenotype of BMDM was identified by detection of CD11b and F4/80 using flow cytometry. The activation phenotype was analyzed by standard RT-PCR for the expression of M1 markers (IL-6, TNF-α, iNOS and IL-1β), M2 markers (Arg-1, CD206 and CD209). The expression of M1 marker HLA-DRα and IL-1β was detected by Western blot and ELISA, respectively.

As for polarized macrophages from THP-1 cells, the M0 conditional supernatant (M0S), M1 conditional supernatant (M1S) or M2 conditional supernatant (M2S) was collected from 24 h culture of THP-1 (PMA), THP-1(PMA + IFN-γ + LPS), or THP-1(PMA + IL-4) cells after stimulants (PMA, IFN-γ, LPS or IL-4) were washed away. As for polarized macrophages from RAW264.7 cells, the M0 conditional supernatant (M0S), M1 conditional supernatant (M1S) or M2 conditional supernatant (M2S) was collected from 24 h culture of RAW264.7, RAW264.7(LPS) or RAW264.7 (IL-4) cells. As for polarized macrophages from BMDMs, the M0 conditional supernatant (M0S), M1 conditional supernatant (M1S) or M2 conditional supernatant (M2S) was collected from 24 h culture of BMDMs, BMDMs (LPS) or BMDMs (IL-4). The human HCC cells (BEL-7402, SMMC-7721 and Huh7) were cultured with medium, M0S, M1S or M2S prepared from polarized THP-1 cells for 6 or 24 h. The murine HCC cell Hepa 1-6 was cultured with medium, LPS, IL-4, M0S, M1S, or M2S prepared from polarized RAW264.7 cells or BMDMs for 6 or 24 h. The 6 h cultures were subjected to RT-PCR assay for PD-L1 mRNA expression while the 24 h cultures were applied to flow cytometry assay for PD-L1 protein expression.

To confirm the responsibility of the inflammatory cytokines for the expression of PD-L1 in HCC cells, receptor blockade assay and cytokine treatment were performed. Huh7 cells were pretreated with IL-1 receptor antagonist (IL-1Ra) (500 ng/ml, 200-01RA, Peprotech, UK) for 30 min, then, stimulated with M1S for 6 h. The PD-L1 expression was detected by quantitative RT-PCR. HCC cells were stimulated with IL-1β (50 ng/ml, 200-01B, Peprotech, UK) for 6 h or 48 h. The expression of PD-L1 was detected by quantitative RT-PCR or flow cytometry.

### siRNA Assay

To identify the cytokines and transcription factors responsible for the PD-L1 expression in HCC cells induced by M1-like macrophages, siRNA assay was performed. The siRNAs targeting IL-1 receptor type 1 (IL1R1), IFN-α receptor type 2 (IFNAR2), IFN-γ receptor type 1 (IFNGR1), P65 or IRF1 were synthesized by Sigma-Aldrich. The target siRNAs and negative control siRNA are listed in Supplementary Table 1. All the siRNAs were transfected into HCC cells using Interferrin (PolyPlus, France). Forty-eight hours after the transfection, the cells were cultured with medium, M1S or IL-1β for 6 h, then, subjected to quantitative RT-PCR assay to quantify the PD-L1 expression.

### RT-PCR

Total RNA was extracted and reversely transcribed into cDNA. Standard RT-PCR was carried out with primers listed in Supplementary Table 2. The PCR products were electrophoresed and the density of the electrophoresis bands was quantified by densitometry with GelPro 3.2 software. The density ratio of each gene relative to β-actin represents its mRNA amount in each individual sample. Quantitative PCR was performed using 2 x SYBR Green qPCR Mix (Aidlab Biotech, China) with specific primers (Supplementary Table 2). The β-actin gene served as an internal control. Relative expression of the target genes to the internal control genes was calculated using the formula: relative expression = 2^−ΔCT^ (ΔCT = CT _targetgene_ – CT _internalcontrol_) (13, 14).

### Western Blot Assay

After electrophoresis of protein extracted from the macrophages, the protein was transferred onto a polyvinylidene difluoride membrane and probed with primary antibodies (anti-HLA-DRa, 2741-1, Epitomics, USA; anti-β-actin, PR-0255, ZSGB-BIO, China) and HRP-conjugated secondary antibodies (goat anti-rabbit IgG antibody, ZB-2301, ZSGB-BIO, China, or goat anti-mouse IgG antibody, ZB-2305, ZSGB-BIO, China). The expression level of each protein was detected by enhanced chemiluminescence system (Beyotime Biotechnology, China).

### Flow Cytometry

The expression of PD-L1 protein in HCC cells, the expression of CD11b and F4/80 in BMDM were quantified by flow cytometry. The cells were harvested and stained with anti-PD-L1 PE (329706, Biolegend, USA), anti-CD11b (101205,Biolegend, USA), anti-F4/80 (565410, Biosciences, USA), mouse IgG1 PE (isotype control, 12-4714-42, eBioscience, USA) for 40 min at 4°C. Then, the cells were detected by flow cytometry. The data were analyzed by Flowjo Software.

### ELISA

The concentration of IL-1β in medium, M1S or M2S was detected by Human IL-1β ELISA Kit (DKW12-1012-096, DAKEWE, China) according to the manufacture's instruction.

### Chromatin Immunoprecipitation (CHIP) Assay

To identify the transcription factors in the PD-L1 promoter region, CHIP assay was carried out. The binding sites of P65 and IRF1 were predicted based on previous studies (15–17) and Jaspar database (http://jaspar.genereg.net/) (Supplementary Figure 2). The BEL-7402 cells were cultured with medium or M1S for 6h. Then, the chromatin from the cells was obtained using SimpleChIP® Enzymatic Chromatin IP Kit (9003,CST,USA) and immunoprecipitated using antibody anti-p65 (8242s, CST, USA), anti-IRF1(ab26109, Abcam, UK) or control IgG (2729P, CST, USA). After purification, the precipitated DNA and input DNA was amplified by quantitative PCR with the specific primers (Supplementary Table 3). The ratios of PCR products from the precipitated DNA to the input DNA represent the relative DNA content conjugated with the antibodies. The ratio of the relative DNA content conjugated with each specific antibody to that conjugated with the control IgG represent occupancy of the corresponding transcription factors in the PD-L1 promoter.

### Site-Directed Mutagenesis and Dual-Luciferase Reporter Assay

To determine the direct binding of transcription factors to the PD-L1 promoter region, the promoter region and the transcription factor binding site mutants were constructed into plasmid pGL3-Basic (Promega, USA). The recombinant vector including the promoter region was constructed and designated as pGL3-PD-L1 in our previous studies (18). Based on this vector, reporter plasmids containing mutation of binding sites of transcription factors were constructed using KOD-Plus-Mutagenesis Kit (TOYOBO, Japan) and designated as pGL3-mut-p65A, pGL3-mut-p65B, pGL3-mut-p65A+B and pGL3-mut-IRF1. The primers used in the site-directed mutagenesis assay are listed in Supplementary Table 4.

BEL-7402 cells were co-transfected with the mixture of luciferase reporter plasmids and Renilla luciferase plasmid pRL-TK (internal control) by Lipofectamine 2000 for 24 h and then were stimulated with M1S for 12 h. The cells were lysed and Luciferase activities were measured with Dual-Luciferase Reporter Assay System (Promega, USA).

### Statistical Analysis

Statistical analysis of the experimental data was performed using GraphPad Prism software (version 6.0). The data is presented as mean ± standard deviation. The difference in the number of CD68^+^ HLA-DR^+^ macrophages between PD-L1 positive regions and PD-L1 negative regions was analyzed by Mann-Whitney test. Student's unpaired *t*-test was used to analyze the difference between two experiment groups in the RT-PCR, flow cytometry, ELISA, CHIP, or dual-luciferase reporter assay. Correlations analysis was carried out using Spearman's rank test. A probability level of 0.05 was considered to indicate a significant difference.

## Results

### Infiltration of M1-Like Macrophages Was Associated With PD-L1 Expression in Human HCC Tissues

**Figure 1:**
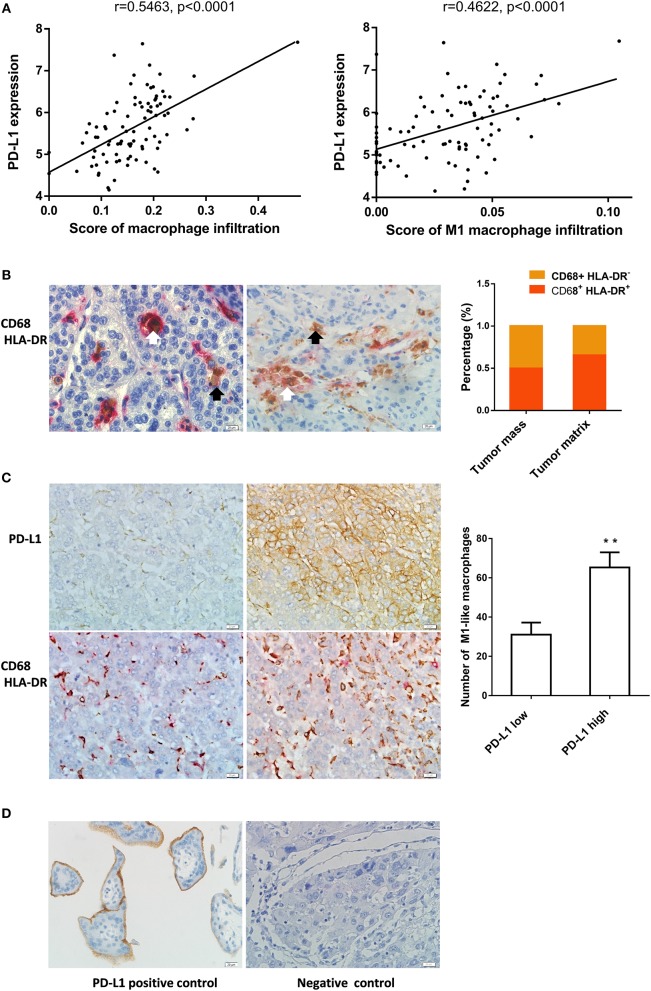
M1-like macrophages infiltration was associated with PD-L1 expression in HCC cells **(A)** The relationship between enrichment of macrophages or M1 macrophages and PD-L1 expression in HCC samples from GEO database. **(B)** The distribution of CD68^+^ HLA-DR^+^ M1 macrophage in HCC tissue. Double immunohistochemical staining was performed by using anti-CD68 and anti-HLA-DR. The black arrow points to CD68^+^ macrophages while the white arrow points to CD68^+^ HLA-DR^+^ M1-like macrophages. **(C)** The association of PD-L1 expression with infiltration of M1 macrophages in HCC tissues. PD-L1 expression and M1-like macrophage distribution were detected in serial sections from the same HCC paraffin tissue. The number of CD68^+^ HLA-DR^+^ macrophages in the PD-L1^+^ region was higher than that in PD-L1^−^ region. **(D)** Positive and negative controls. A placental chorionic tissue was used as positive control (left). Negative controls were performed by omitting the primary antibodies (right). The data are presented as mean±SD. ** *p* < 0.01.

In order to reveal the association between M1 macrophages and PD-L1 expression in HCC tissues, we analyzed enrichment of macrophage or M1 macrophage in 90 HCC samples from GEO database using the webtool xCell. We found that the enrichment scores of macrophages or M1 macrophages was correlated to PD-L1 expression in the HCC tissues (Figure 1A). Besides, the enrichment score of M2 macrophages or CD8+ T cells was also correlated to PD-L1 expression (Supplementary Figure 5).

Next, we analyzed the relationship between infiltration of M1 macrophage in HCC tissues and PD-L1 expression in HCC cells using immunochemistry assay. The CD68^+^ HLA-DR^+^ M1-like macrophages were found in 58 cases of HCC specimens. The CD68^+^ HLA-DR^+^ M1-like macrophages and CD68^+^ macrophages were counted in 40 tumor mass regions and 40 tumor matrix regions selected from these HCC specimens. The percentage of CD68^+^ HLA-DR^+^ M1 macrophages in tumor mass was 50.2% while that in tumor matrix was 65.5% (Figure 1B). Fifty-four cases of the HCC specimens were examined for the PD-L1 expression in HCC cells and 8 cases were PD-L1 positive. Eighteen PD-L1 positive regions where the HCC cells expressed PD-L1 protein (≥10 PD-L1^+^ cells / high power field) and eighteen PD-L1 negative regions where the PD-L1 expression is negative or low (<10 PD-L1^+^ cells / high power field) were selected from the PD-L1 positive cases. The number of CD68^+^ HLA-DR^+^ macrophages in the corresponding region of the serial sections was counted. We found that the number of CD68^+^ HLA-DR^+^ macrophages in the PD-L1 positive regions was higher than that in the PD-L1 negative regions (Figure 1C). The anti-PD-L1 used in the immunohistochemistry assay is specific to the PD-L1 molecule since expression of PD-L1 was detected in placental chorionic tissues (Figure 1D). These data demonstrate that PD-L1 expression in HCC cells was correlated to infiltration of M1-like macrophages.

### M1 Macrophages Induced PD-L1 Expression in HCC Cells

**Figure 2:**
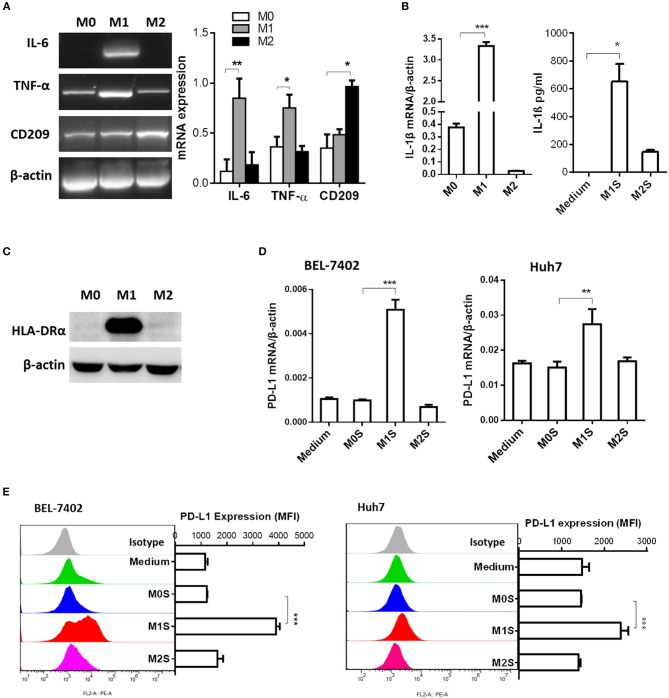
M1 macrophages derived from THP-1 cell upregulated expression of PD-L1 in human HCC cells. THP-1 cells were treated by PMA for 6h, then activated by LPS or IL-4 for 18h. THP-1 (PMA), THP-1(PMA + IFN-γ + LPS), or THP-1(PMA + IL-4) was polarized into macrophages with M0, M1 or M2 phenotype. **(A)** Expression of IL-6, TNF and CD209 was quantified by RT-PCR. **(B)** Expression of IL-1β mRNA was determined by quantitative RT-PCR while expression of IL-1β protein in M1S or M2S was examined by ELISA assay. **(C)** HLA-DRα expression was detected by Western blot assay. **(D)** PD-L1 expression in HCC cells treated with medium, M0S, M1S, or M2S was determined by quantitative RT-PCR. **(E)** PD-L1 expression in HCC cells treated with medium, M0S, M1S, or M2S was determined by flow cytometry. The experiment was carried out in triplicate and repeated at least twice. Data are shown as means ± SD. *P < 0.05, **P < 0.01, ***P < 0.001.

To clarify the causal relationship between M1 macrophages and PD-L1 expression in HCC cells, we performed *in vitro* experiments using M1 macrophages derived from THP-1 cells. Phenotype of M0, M1 or M2 was detected in THP-1 (PMA), THP-1(PMA + IFN-γ + LPS), or THP-1(PMA + IL-4) cells. The M1 phenotype was marked by elevated expression of IL-6, TNF-α (Figure 2A), IL-1β (Figure 2B) and HLA-DRα(Figure 2C). The M2 phenotype was marked by elevated expression of CD209. Then, the M0S, M1S, or M2S was added to the culture of HCC cell line BEL-7402 or Huh 7. The results showed that M1S induced expression of PD-L1 in HCC cells at both mRNA and protein levels instead of M2S (Figures 2D,E).

**Figure 3:**
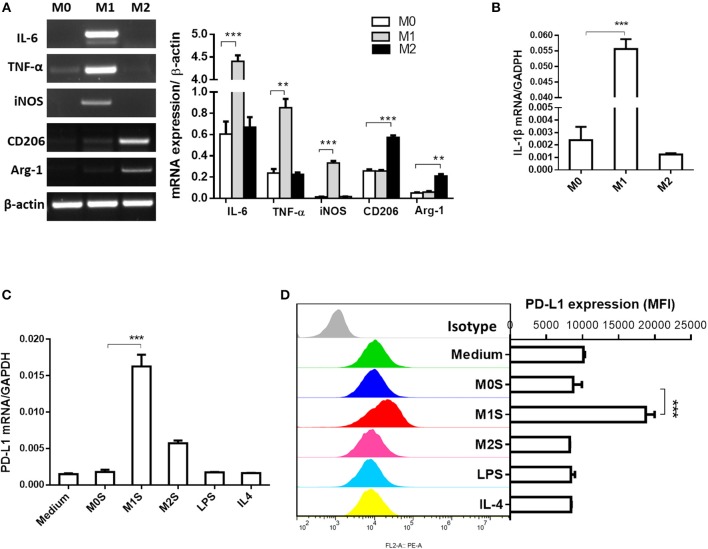
M1 macrophages derived from RAW264.7 cell upregulated expression of PD-L1 in murine HCC cells. RAW264.7 cells were stimulated by LPS or IL-4 and polarized into RAW264.7(LPS) or RAW264.7 (IL-4) cells with M1 or M2 phenotype, respectively. RAW264.7 cells without stimulation were defined as M0. **(A)** Expression of IL-6, TNF-α, iNOS, CD206 and Arg-1 was detected by RT-PCR. **(B)** Expression of IL-1β was determined by quantitative RT-PCR. **(C)** PD-L1 mRNA expression in Hepa 1-6 cells treated with medium, M0S, M1S, M2S, LPS, or IL-4 was quantified by quantitative RT-PCR. **(D)** PD-L1 expression at protein level in Hepa 1-6 cells treated with medium, M0S, M1S, M2S, LPS, or IL-4 was determined by flow cytometry. Similar results were obtained in two independent experiments performed in triplicate. Data are shown as means ± SD. **P < 0.01, ***P < 0.001.

M1 macrophages derived from RAW264.7 cells induced expression of PD-L1 in murine HCC cells. Phenotype of M0, M1, or M2 was evaluated in RAW264.7, RAW264.7(LPS) or RAW264.7 (IL-4) cells. Expression of M1 phenotype markers (IL-6, TNF-α, iNOS and IL-1β) was high in the RAW264.7(LPS) cells and expression of M2 phenotype markers (CD206 and Arg-1) was elevated in the RAW264.7(IL-4) cells, compared to the M0 macrophages (RAW264.7) (Figures 3A,B). The M1S but not M2S derived from the polarized RAW264.7 cells dramatically induced the expression of PD-L1 in HCC cells (Figures 3C,D).

**Figure 4:**
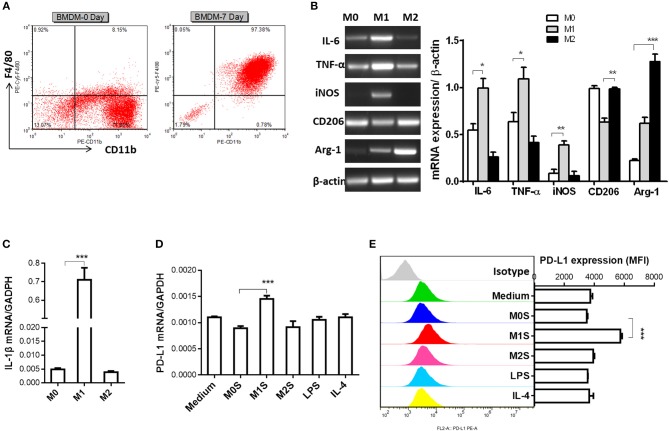
M1 macrophages derived from BMDM upregulated expression of PD-L1 in murine HCC cells**. (A)** Bone marrow cells from C57BL/6 mouse were cultured with M-CSF for 7 days. Flow cytometry assay was performed to identify mature BMDM by using F4/80 and CD11b as identification markers. **(B)** BMDMs were stimulated by LPS or IL-4 for 12 h to generate BMDMs (LPS) or BMDMs (IL-4) with M1 or M2 phenotype, respectively. The BMDMs without stimulation were defined as M0. Expression of IL-6, TNF-α, iNOS, CD206 and Arg-1 was detected by RT-PCR. **(C)** Expression of IL-1β was determined by quantitative RT-PCR. **(D)** PD-L1 mRNA expression in the Hepa 1-6 cells treated with medium, M0S, M1S, M2S, LPS or IL-4 was quantified by RT-PCR assay. **(E)** PD-L1 protein expression in the Hepa 1-6 cells treated with medium, M0S, M1S, M2S, LPS, or IL-4 was quantified by flow cytometry. The experiment was carried out in triplicate and repeated at least twice. Data are shown as means ± SD. *P < 0.05, **P < 0.01, ***P < 0.001.

M1 macrophages derived from murine bone marrow promoted expression of PD-L1 in murine HCC cells. In order to generalize our findings, BMDMs were used to prepare M1 macrophages. The mature macrophages were generated from murine bone marrow with 97.38% F4/80^+^CD11b^+^ BMDMs (Figure 4A). The M1 phenotype of BMDMs (LPS) was marked by elevated expression of IL-6, TNF-α, iNOS and IL-1β. The BMDMs (IL-4) displayed increased expression of CD206 and Arg-1 compared with the M0 macrophages (BMDMs) (Figures 4B,C). Then, we investigated whether the M1 macrophages derived from BMDMs can upregulate PD-L1 expression in murine HCC cells. The results showed that PD-L1 expression in the HCC cells treated with the M1S was increased, but not in the HCC cells treated with the M2 (Figures 4D,E). All these results indicate that M1 macrophages significantly promoted PD-L1 expression in HCC cells at both mRNA and protein level.

### M1 Macrophages Induced the PD-L1 Expression in HCC Cells Through IL-1β Signaling

**Figure 5:**
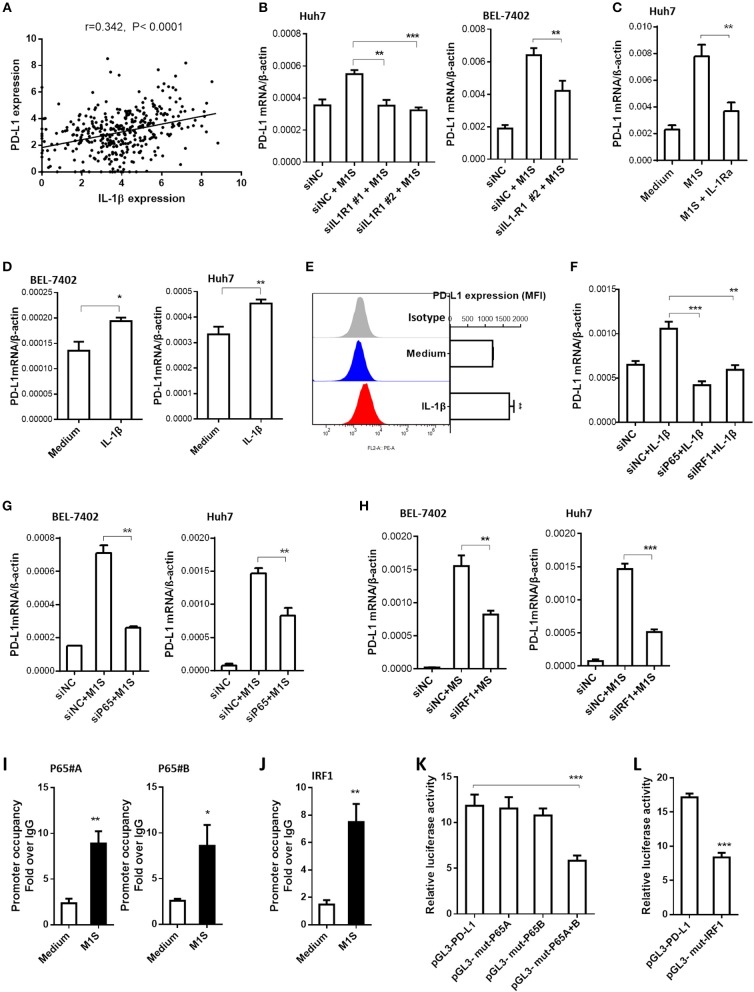
IL-1β signaling was responsible for PD-L1 expression in HCC cells induced by M1 macrophages. **(A)** Co-expression of IL-1β and PD-L1 gene in HCC samples from TCGA database. **(B)** HCC cells were transfected with siNC or siIL-1R1, then cultured in the presence or absence of M1S. The PD-L1 expression in these HCC cells was determined by quantitative RT-PCR assay. **(C)** Huh7 cells pretreated with IL-1Ra were cultured in the presence or absence of M1S. The PD-L1 expression was detected by quantitative RT-PCR. **(D)** HCC (BEL-7402 or Huh7) cells were stimulated with IL-1β. The PD-L1 expression was detected by quantitative RT-PCR. **(E)** Huh7 cells were stimulated with IL-1β. The PD-L1 expression was detected by flow cytometry. **(F)** Huh7 cells were transfected with siNC, siP65, or siIRF1, then cultured in the presence or absence of IL-1β. The PD-L1 expression was determined by quantitative RT-PCR assay. **(G,H)** HCC cells were transfected with siNC, siP65, or siIRF1, then cultured in the presence or absence of M1S. The PD-L1 expression was determined by quantitative RT-PCR assay. **(I,J)** BEL-7402 cells were treated with or without M1S, then, CHIP assay was performed to assess the occupancy of transcription factor P65 or IRF1 on the PD-L1 promoter. **(K,L)** BEL-7402 cells were co-transfected with the mixture of internal control plasmid pRL-TK and luciferase reporter plasmid pGL3-PD-L1, pGL3-mut-p65A, pGL3-mut-p65B, pGL3-mut-p65A+B, or pGL3-mut-IRF1, then treated with M1S. The relative luciferase activities were measured by dual-luciferase reporter assay. The experiment was carried out in triplicate and repeated at least twice. Data are shown as means ± SD. *P < 0.05, **P < 0.01, ***P < 0.001.

As the IL-1β is an important proinflammatory cytokine secreted by M1 macrophages, we analyzed the relationship between IL-1β expression and PD-L1 expression in HCC tissues using the webtool muR2. The results showed that expression of PD-L1 was correlated to expression of IL-1β in 371 HCC samples from the TCGA database (Figure 5A).

Subsequently, we designed siRNA assay or cytokine receptor blockade assay to investigate whether the PD-L1 expression in HCC cells was promoted by the cytokine IL-1β. The effectiveness of siRNAs targeting IL-1β receptor was determined by quantitative RT-PCR (Supplementary Figure 3c). Knockdown of IL-1β receptor inhibited the PD-L1 expression induced by M1 macrophages (Figure 5B). These results suggest that the cytokine IL-1β was responsible for the inducible PD-L1 expression in HCC cells. The role of IL-1β was confirmed further by IL-1 receptor blockade assay using IL-1Ra (Figure 5C). Since IFN type I and IFN type II can induce the expression of PD-L1 in cancer cells, we also use siRNAs targeting IFNAR2 or IFNGR1 to silence expression of IFNAR2 or INFGR1 in HCC cells (Supplementary Figures 3A,B). But the knockdown of these IFN receptors did not affect the induction of PD-L1 expression in HCC cells by M1 macrophages (Supplementary Figure 4). These results suggested that IFN type I and type II were not responsible for the inducible expression of PD-L1. To further confirm the responsibility of proinflammatory cytokine IL-1β, HCC cells were stimulated with recombinational IL-1β. The results showed that the recombinational cytokine induced PD-L1 expression in HCC cells (Figures 5D,E).

The proinflammatory cytokines engage their receptors on the HCC cells to elicit signaling transduction, leading to changes of gene expression. IRF1 and NF-κB are dominant transcription factors at the terminal of IL-1 signaling (19). Thus, we hypothesized that M1 macrophages promoted PD-L1 expression in HCC cells depending on NF-κB and IRF1. In order to support this hypothesis, we performed siRNA assay using siRNA targeting p65 and IRF1. The results showed that knockdown of expression of these transcription factors inhibited PD-L1 expression induced by M1S or IL-β (Figures 5F–H).

Next, we asked whether these transcription factors were located on the PD-L1 promoter region when the PD-L1 expression was initiated by M1S. The results from the CHIP assay showed that occupancy of these transcription factors in the PD-L1 promoter region was increased when the HCC cells were cultured in M1S (Figures 5I,J). These results suggest accumulation of these transcription factors in the PD-L1 promoter region. To further determine whether these transcription factors directly bind to their corresponding binding sites, we performed site-directed mutagenesis assay. Based on reporter plasmid pGL3-PD-L1, the reporter plasmids containing mutation (pGL3-mut-p65A, pGL3-mut-p65B, pGL3-mut-p65A+B and pGL3-mut-IRF1) were constructed. Dual-luciferase reporter assay was performed with theses vectors in HCC cells cultured in M1S. The results showed that combinatorial mutation in the two p65 binding sites attenuated the luciferase activity induced by M1S (Figure 5K). So did the mutation in the IRF1 binding site (Figure 5L). These results demonstrate that p65 and IRF1 directly bound to the PD-L1 promoter.

## Discussion

Macrophages are critical constituents in the inflammatory environment of HCC. Although M2 macrophages play a pro-tumor role, the relevance of M1 macrophages to HCC development is unclear. In the present study, we found that there was an association between infiltration of M1-like macrophages and PD-L1 expression in HCC tissues. Moreover, M1 macrophages secreted IL-1β to induce PD-L1 expression in HCC cells. Besides, the inducible expression of PD-L1 depended on transcription factors IRF1 and NF-κB. All these findings suggest that M1 macrophages may play a pro-tumor role by promoting the expression of PD-L1 in HCC cells.

The role of M1 macrophages is double-edged since these cells have both anti-tumor and pro-tumor functions. On one hand, M1 macrophages are an important effector in immune surveillance (20). M1 macrophage infiltration is associated with good prognosis in some cancers (21, 22). M1 macrophages inhibit proliferation or induce apoptosis of cancer cells (13, 14). As for HCC, low M1 and high M2 macrophages are associated with poor prognosis of HCC patients (23). On the other hand, M1 macrophages can promote the progression of cancers. For example, M1 macrophages are protumorigenic in early stage of urethane-induced lung carcinogenesis (24). In addition, infiltration of HLA-DR+ M1 macrophages indicates a poor response to radiotherapy for patients with rectal cancers (25). Moreover, M1 macrophages can play a pro-metastatic role since they induce EMT changes in pancreatic cancer cells (26) and HCC cells (6). Additionally, the PD-L1 expressed on the M1-like TAM results in immune escape of HCC (27). Our results showed that macrophages infiltration correlated to PD-L1 expression in HCC cells. These results are in line with findings in a previous study investigating relationship between tumor microenvironment and PD-L1 expression in malignant pleural mesotheliomas. The study showed that infiltration of macrophages was associated with PD-L1 expression in malignant cells (28). We further found that M1 macrophages induced expression of PD-L1 in HCC cells by *in vitro* experiments (But the immunosuppressive role of M1 macrophages remains to be demonstrated by an *in vivo* experimental model). The Janus-faced role of M1 macrophages raises the question of the optimal strategy for targeting macrophages in anticancer therapy. Tumor immunotherapeutic strategy of re-polarizing M2 TAM into M1 phenotype may be less effective and should be regarded with caution. More promising and effective strategies seem to be combination of M1 macrophages polarization therapies with PD-L1/PD-1 checkpoint blockade.

We found that cytokine IL-1β secreted by M1 macrophages induced PD-L1 expression via transcription factor p65 and IRF1 in HCC cells. The IL-1β engages its receptors IL-1R1 and IL-1 receptor accessory protein (IL-1RacP) on the target cells. The engagement leads to cytosolic regions of these receptors called Toll- and IL-1R–like (TIR) domains brought into the proximity to each other. These TIR domains recruits adaptor protein MyD88, which initiates the cytosolic signaling resulting in activation of transcription factor NF-κB (29). The activation of NF-κB induces expression of E3 ligase IAP2, which mediates K63-linked ubiquitination of IRF1. The K63-linked ubiquitination is a prerequisite for the activity of IRF1 (30). NF-κB and IRF1 are critical for transcription of PD-L1 genes since binding sites for NF-κB subunit p65 and IRF1 are present in the promoter of PD-L1 gene (16, 17, 31). Hence, the IL-1β signaling is the underlying mechanism for the M1 macrophages to induce PD-L1 expression in HCC cells. Our findings provided therapeutic targets for blockade of the inducible PD-L1 expression. Some agents targeting IL-1β, such as IL-1 receptor antagonist, soluble decoy IL-1 receptor and neutralizing monoclonal antibodies against IL-1, have been approved in clinics to treat autoimmunity or inflammatory diseases (32, 33). Therefore, further investigation should be carried out to observe whether these drugs may serve as adjuvants in HCC treatment to attenuate the induction of PD-L1 expression by M1 TAM.

Our findings that the PD-L1 expression in HCC cells was induced by M1 macrophages partially explain how the inducible PD-L1 expression in cancer cells occurs. The most potent inducer for PD-L1 expression in a variety of cancers is IFN-γ (10). A theory referred as adaptive resistance is proposed to explain this phenomenon. Tumor-specific CTL infiltrated in the tumor tissues releases IFN-γ, which in turn induce cancer cells to express PD-L1. The induced PD-L1 engages its receptor PD-1 expressed on the CTL to inhibit the anti-tumor immunity mediated by the CTL (34, 35). Our results show that the inflammatory cytokine IL-1β secreted by M1 macrophages induce expression of PD-L1 in HCC cells. Besides, previous studies demonstrate the PD-1 is not only expressed in T cells but also on activated macrophages (36). The PD-1 signaling can induce apoptosis (37) or suppression of phagocytosis of the macrophages (38). Consequently, it can be speculated that the inducible PD-L1 expression in HCC cells may reacts with the PD-1 on the macrophages, leading to inhibition of the macrophage-mediated anti-tumor immunity. Thus, the macrophage-induced PD-L1 expression may also be regarded as adaptive resistance of tumor cells to the anti-tumor immunity. Therefore, our findings provide evidence to complement the adaptive resistance theory.

There are some limitations in the present study. First, the macrophages used in this study were derived from cell lines or murine BMDM. They may be different from M1-like TAM in HCC tissues. Second, there is no document about prognosis of the HCC patients in the present study although a previous study showed that PD-L1 expression on HCC cells was negatively associated with patients' overall survival (23). Third, it was not investigated whether the PD-L1 expression induced in HCC cells by M1 macrophages leads to immune escape of the HCC. The inhibitory effect of the induced PD-L1 expression on CTL- or macrophage-mediated anti-tumor immunity remains to be researched in our next work. In addition, SYBR qPCR Master Mix was used in the quantitative RT-PCR assay to determine expression of PD-L1 in HCC cells instead of Taqman Gene Expression Master Mix. This may lead to decreased detection sensitivity for the PD-L1 expression.

In summary, we found an association between infiltration of M1 macrophages in HCC tissues and PD-L1 expression in HCC cells. M1 macrophages derived from THP-1 or RAW264.7 cell lines, as well as murine BMDM, induced PD-L1 expression in human or murine HCC cells. The inflammatory cytokine IL-1β secreted by the M1 macrophages is responsible for the induced PD-L1 expression. Transcription factor IRF1and P65 were identified to be involved in the inducible PD-L1 expression. All the data support the pro-tumor role of M1 macrophages and provide therapeutic targets for interruption of the inducible PD-L1 expression in HCC cells.

## Data Availability

Publicly available datasets were analyzed in this study. This data can be found here: https://www.ncbi.nlm.nih.gov/geo/, under accession numbers GSE41804 and GSE121248. The other data supporting the conclusions of this manuscript will be made available by the authors, without undue reservation, to any qualified researcher.

## Ethics Statement

This study was carried out in accordance with the recommendations of Medical Ethical Committee of Shandong University with written informed consent from all subjects. All subjects gave written informed consent in accordance with the Declaration of Helsinki. The protocol was approved by the Medical Ethical Committee of Shandong University.

This study was carried out in accordance with the recommendations of the Animal Ethical and Welfare Committee of Shandong University School of Medicine with the permit number 201302072. The protocol was approved by the Animal Ethical and Welfare Committee of Shandong University School of Medicine.

## Author Contributions

YS designed and supervised the research. ZZ, JZ, and RM performed the cell biological experiments and the animal experiments. NL and CM analyzed and interpreted the patient data. JW and XW contributed reagents. HZ and LZ contributed data analysis and interpretation. ZZ and YS prepared figures and wrote the paper. All authors read and approved the final manuscript.

### Conflict of Interest Statement

The authors declare that the research was conducted in the absence of any commercial or financial relationships that could be construed as a potential conflict of interest.
