# Supplementary material for: M1 Macrophages Induce PD-L1 Expression in Hepatocellular Carcinoma Cells Through IL-1β Signaling
Source: Front Immunol. 2019 Jul 16;10:1643. doi: 10.3389/fimmu.2019.01643 (PMC6648893; doi:10.3389/fimmu.2019.01643)
Supplement: Supplementary file 1 [file Data_Sheet_1.docx]

Supplementary Material

## Supplementary Figures


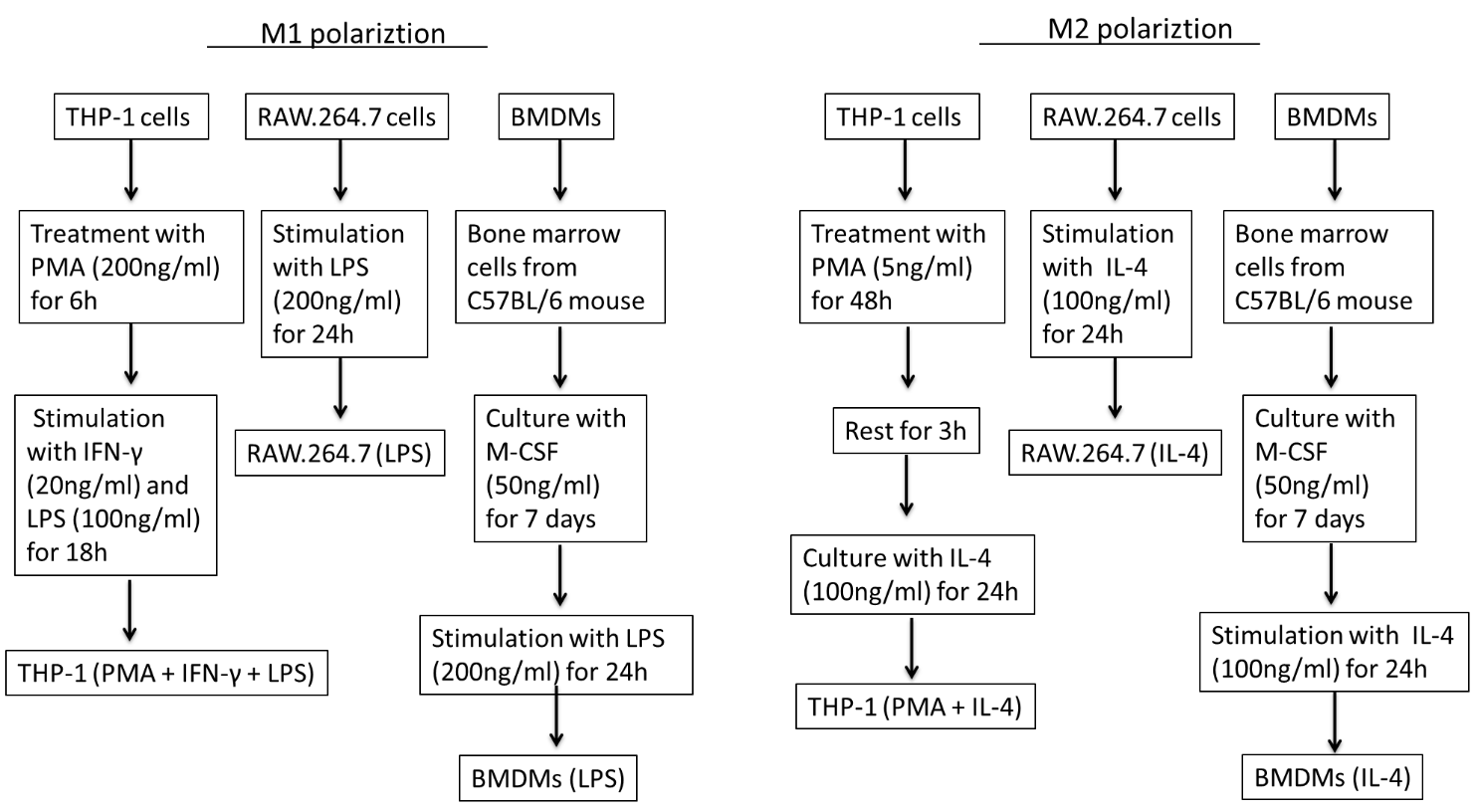


**Figure S1** Schematic diagram of macrophages polarization

As for polarization of THP-1 cells, the M0, M1 or M2 macrophages is designated as THP-1 (PMA), THP-1(PMA + IFN-γ + LPS), or THP-1(PMA + IL-4) cells. As for polarization of RAW264.7 cells, the M0, M1 or M2 macrophages is designated as RAW264.7, RAW264.7(LPS) or RAW264.7 (IL-4) cells. As for polarization of BMDMs, the M0, M1 or M2 macrophages is designated as BMDMs, BMDMs (LPS) or BMDMs (IL-4).


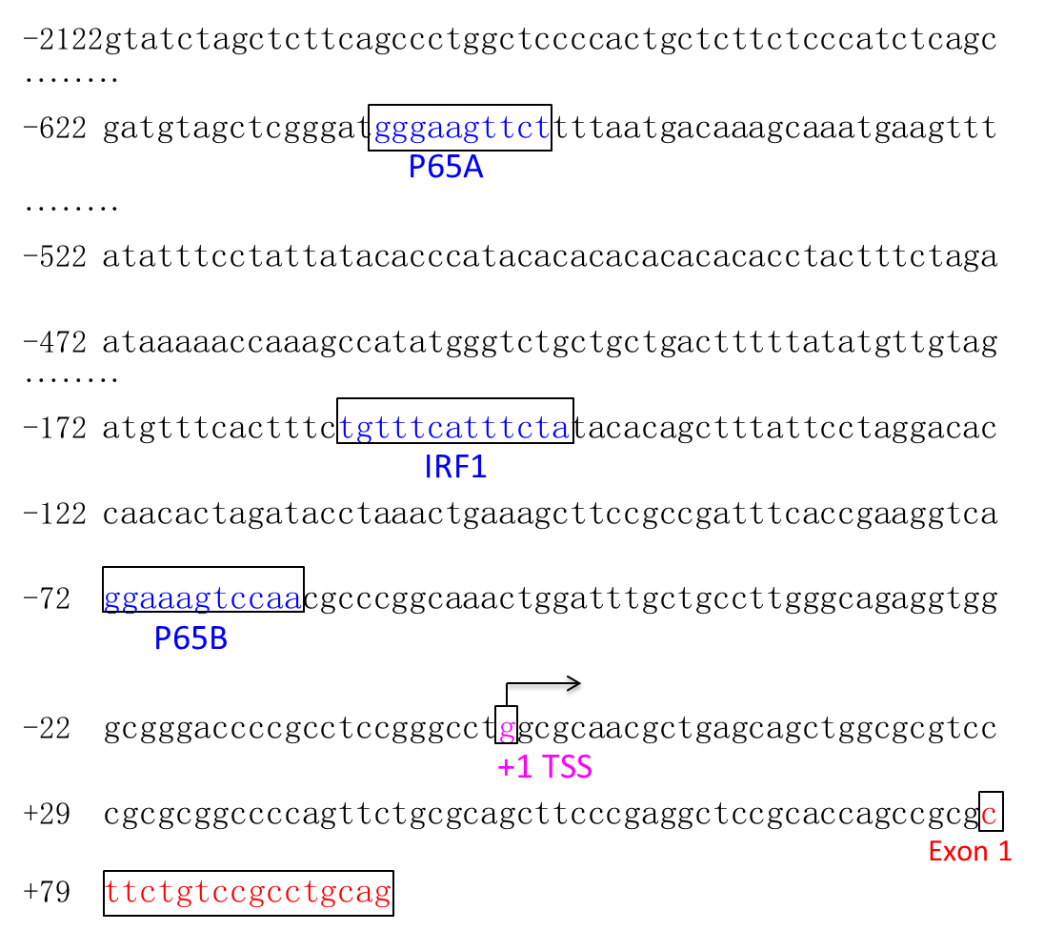


**Figure S2** PD-L1 promoter region analysis

The promoter region (-2122 to +94, TSS was assigned +1) includes two binding sites for p65 (P65A, -607 to -597; p65B, -72 to -62) and one IRF1 binding site (-159 to -147).


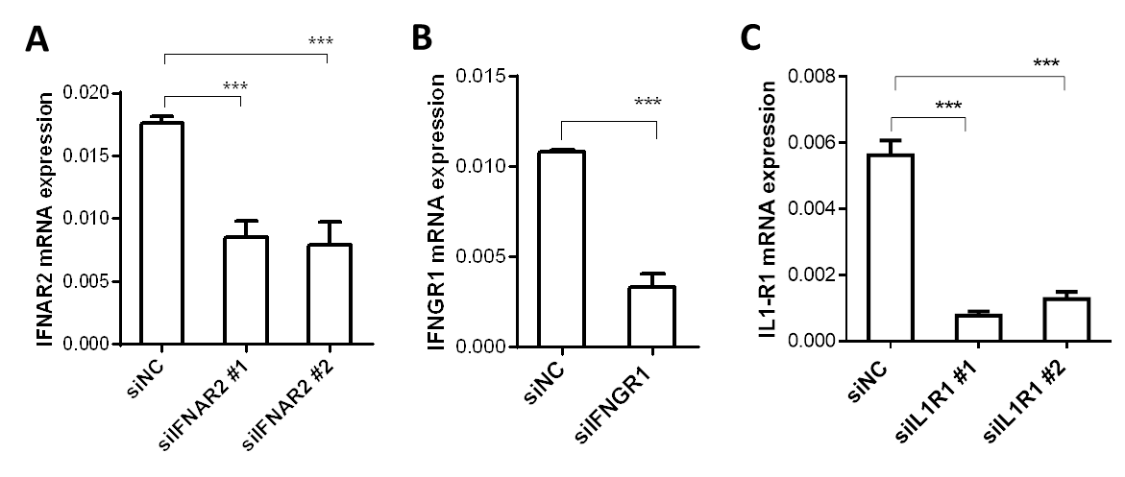


**Figure S3** Efficacy of siRNAs

(A) Huh7 cells were transfected with siRNAs against IFNAR2 for 48h. The expression of IFNAR2 was detected by quantitative RT-PCR. (B) Huh7 cells were transfected with siRNAs against IFNGR1 for 48h. The expression of IFNGR1 was detected by quantitative RT-PCR. (C) Huh7 cells were transfected with siRNAs against IL1R1 for 48h. The expression of IL1R1 was detected by quantitative RT-PCR. Data are shown as means ± SD. **P < 0.01, ***P < 0.001.


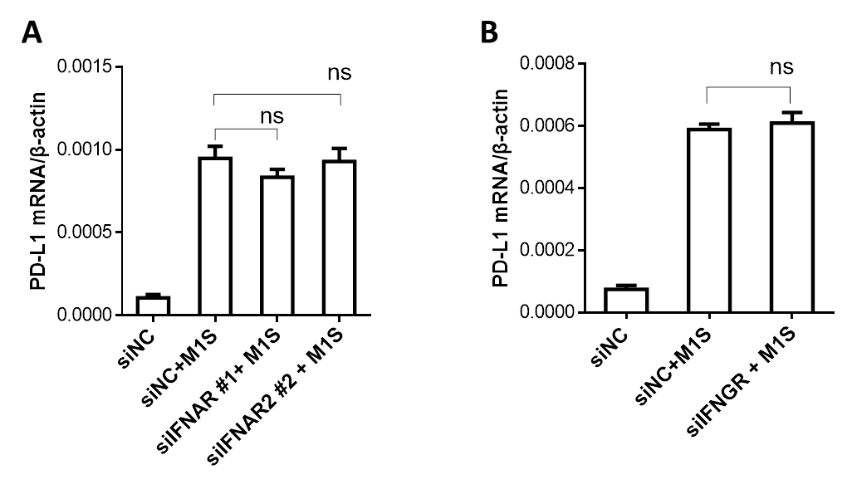


**Figure S4** Knockdown of IFN receptors did not block the induction of PD-L1 expression by M1-like macrophages

(A) Huh7 cells were transfected with siNC or siRNAs against IFNAR2 for 48h, then, treated with M1S for 6h. The PD-L1 expression was detected by quantitative RT-PCR. (B) Huh7 cells were transfected with siNC or siFNGR1 for 48h, then, treated with M1S for 6h. The PD-L1 expression was detected by quantitative RT-PCR. Data are shown as means ± SD. ns, no significance.


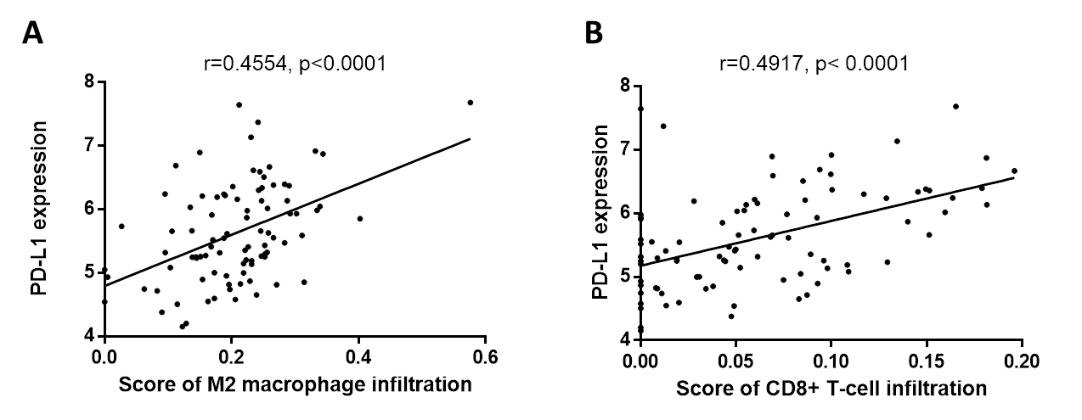


**Figure S5** The relationship between enrichment of CD8+ T cells or M2 macrophages and PD-L1 expression in HCC samples from GEO database.

(A) Score of M2 macrophage infiltration correlated to PD-L1 mRNA expression in HCC tissues. (B) Score of CD8+ T cell infiltration correlated to PD-L1 mRNA expression in HCC tissues.

## Supplementary Table

##
